# Friction Investigation of Closed-Cell Aluminium Foam during Radial-Constrained Test

**DOI:** 10.3390/ma17133344

**Published:** 2024-07-05

**Authors:** Jozsef Kertesz, Tünde Anna Kovacs

**Affiliations:** 1 Department of Vehicle Engineering, Faculty of Engineering, University of Debrecen, 4032 Debrecen, Hungary; 2 Doctoral School on Safety and Security Science, Obuda University, 1034 Budapest, Hungary; 3 Banki Donat Faculty of Mechanical and Safety Engineering, Obuda University, 1034 Budapest, Hungary; kovacs.tunde@bgk.uni-obuda.hu

**Keywords:** friction, closed-cell aluminium foam, compression test, radial constrain, absorbed energy, crashworthiness

## Abstract

The energy-absorbing capacity and friction phenomena of different closed-cell aluminium foam-filled Al tube types are investigated through experimental compression tests. Concerning the kind of investigation, free, radial-constrained and friction tests occurred. The radial-constrained compression test results confirm that the process requires significantly more compression energy than without the constrain. Pushing away different pre-compressed foams inside the aluminium tube, the static and kinematic frictional resistances can be determined and the energy required to move them can be calculated. Knowing the value of the energy required for the frictional resistance, we can obtain how much of the energy surplus in radially inhibited compression is caused by the friction phenomena. The main goal present study is to reveal the magnitude of friction between the foam and the wall of the tube during the radially constrained test. The investigation used 0.4 and 0.7 g/cm^3^ density closed-cell aluminium foam whilst a compressive test was applied where the force–displacement data were recorded to calculate the absorbed energy due to friction. Considering the results of the test, it can be stated that 18% of the invested energy was used to overcome friction in the case of lighter foam and almost 23% with 0.7 g/cm^3^ foam during the radial-constrained test.

## 1. Introduction

Aluminium foams with closed cells are used in structural applications and provide lightweight and impact-absorbing qualities for automobiles [1,2,3,4]. These foam’s mechanical characteristics are shaped by the way their topology and geometry change upon impact. Made with powder metallurgy, these materials have significant energy absorption capacities and are deemed promising for use in a range of sectors [5,6,7]. According to research, vehicle crash boxes filled with aluminium foam have far better energy absorption per unit mass and length, allowing for shorter crash boxes with the same amount of energy absorption [8,9,10,11]. Through the creation of a lightweight, modular composite framework that can be adjusted for impact absorption, this technique improves structural safety in automobiles [12]. Research has investigated the application of foam-filled aluminium tubes in automobile production to improve crashworthiness and maximise lightweight design. Pore structure, cell wall flaws, and cell wall microstructure are some of the elements that affect the mechanical characteristics of closed-cell aluminium foams [13,14,15]. The energy absorption capacity of closed-cell aluminium foam remains almost constant throughout a range of strain rates, but the compressive behaviour exhibits notable strain rate hardening [16,17]. When compared to other cellular metals, the specific energy absorption of closed-cell aluminium foam demonstrates a fair degree of agreement in terms of deformation energy. The mechanical response of closed-cell aluminium foam can now be predicted quickly, reliably, and accurately thanks to the development of a computer model that has been verified by experimental data [18,19,20,21]. The mechanical properties of closed-cell aluminium foams have been extensively studied in both confined and unconstrained situations [22,23,24]. Additionally, the same foams have been tested and placed into tubes. From the point of view of crashworthiness safety development, the main goal of the investigation is to maximalise the energy absorbing capacity of the actual foam, without significant weight increase [25,26]. One of the most improved ways to reveal the absorbing behaviour of the foam is the radial-constrained compression condition. This means the foam is inserted into a thin-walled structure as, for example, the crash box is, and this porous material is compressed inside the inhibitor. The inhibitor could be made from different materials such as aluminium tubes or plates, but the composite is also applied as a constraint [27,28,29]. However, the relationship between the energy surplus and the friction phenomenon occurring during the radial force test is not detailed in the literature.

Due to the surface complexity of a closed-cell foam, it is hard to define its surface roughness and its friction conditions. The size of the cells has an effect not only on the energy-absorbing capacity but also on the friction between the foam and the connected surface [30]. One important component of material behaviour that affects the functionality and uses of closed-cell aluminium foams is friction [31,32,33,34]. However, there was no relevant information about friction inside of the Al tube during the radial-constrained compression test. Therefore, the main goal of the present research was to reveal the magnitude of Coulomb friction during a radial-constrained test of closed-cell aluminium foam, for which a complex friction measurement method was used. This study is part of more complex research, namely the enhancement of vehicle crash safety with structural solutions, where the goal is to create a multi-membered crash box structure filled with metal foam. The results detailed here significantly help our further development work, and hopefully provide useful and inspiring information on the science of crashworthiness with metal foam.

## 2. Materials and Experimental Methods

### 2.1. Preparation of Specimens

**Figure 1:**
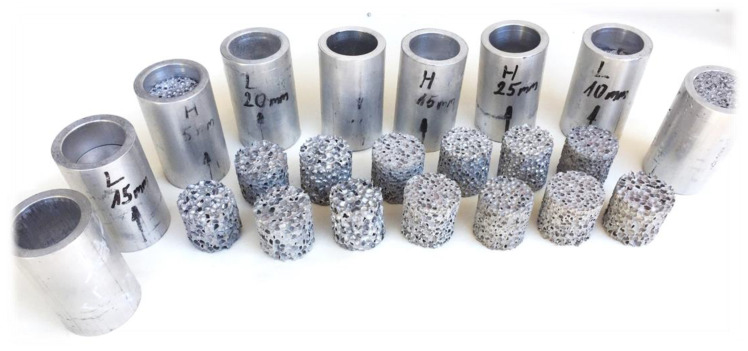
Specimens for the test.

The present research focuses on the energy-absorbing behaviour of closed-cell aluminium foam to which the base material was manufactured by Behai Composite Materials Co. (Jiujiang, China) in the form of 600 × 600 × 30 mm blocks with two different densities, numerically 0.4 and 0.7 g/cm^3^ [35]. The chemical ingredients of the foam were as follows: 97% aluminium powder, 3% foaming agent, 1% TiH_2_, increasing viscose, and 2% Ca. They are environmental protection elements that have no pollution. For the compressive test, 30 mm diameter and height specimens were made using a waterjet cutting process. Owing to the precise cutting process, the specimens had a consistent height and diameter which were measured using a digital calliper at more points along the foam’s height. To not influence the test results by the possible inner material or structure issues of foam specimens, all of the measurements were repeated three times to increase the statistical meaning, but the later results are around the mean of values. During the radially constrained investigation, the foam specimens were inserted into a 30 mm inner diameter aluminium tube with a wall thickness of 5 mm and 65 mm height and compressed using a pushing plate with a 28 mm nominal diameter and 3 mm thickness. The tube preparation did not require any type of surface finishing or machining over the cutting up with 65 mm. Considering the material of the tube and pushing plate, the AlMgSi AW EN6061 was applied as one of the frequently used materials in the field of automotive construction, mainly in the bumper system and its accessories. Between the test machine crosshead and the pushing plate, a so-called push rod was inserted in the form of a 28 mm nominal diameter tube. The prepared specimens before the test are presented in Figure 1, whilst their geometry data are collected in Table 1. According to the dimensions and weights, the theoretical densities are confirmed.

### 2.2. Test Conditions and Tools

**Figure 2:**
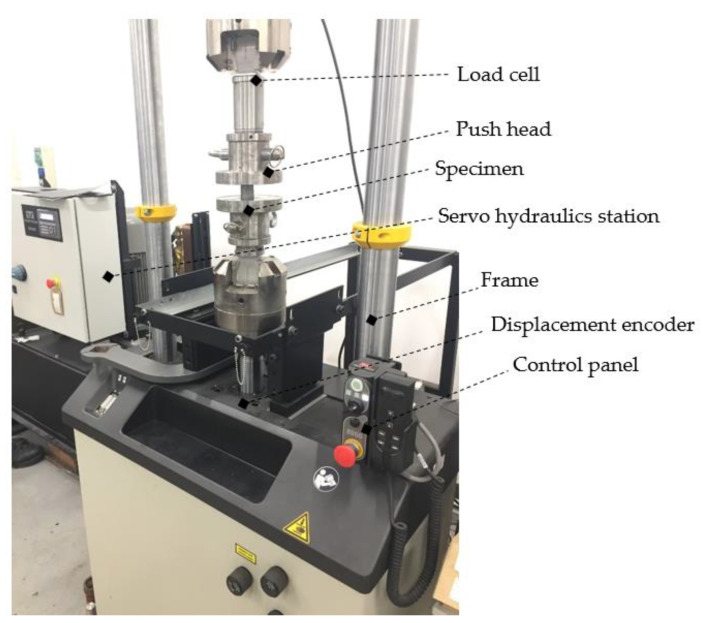
Tools for investigation.

For the test, a INSTRON 8801 servo hydraulics material fatigue test machine was used, connected with a data collector and analysis software, namely WaveMatrix v.2, to record the force and displacement values. The velocity of the crosshead of the test machine was constant at 1 mm/s, whilst the system data recording frequency was 1000/s. The displacement is recorded using an implement displacement encoder, and the force is detected using a load cell with a limit of 100 kN. The measurement was carried out in an accredited laboratory and the measuring instrument had a valid calibration and verification logbook at the time of measurement. Before the test, owing to the prevention of technical integrity of the load cell, the 80 kN maximum force limit was adjusted; if this condition was exceeded, the operation was immediately blocked. The components of the test machine are in Figure 2.

### 2.3. Scheme of Compressive Tests

**Figure 3:**
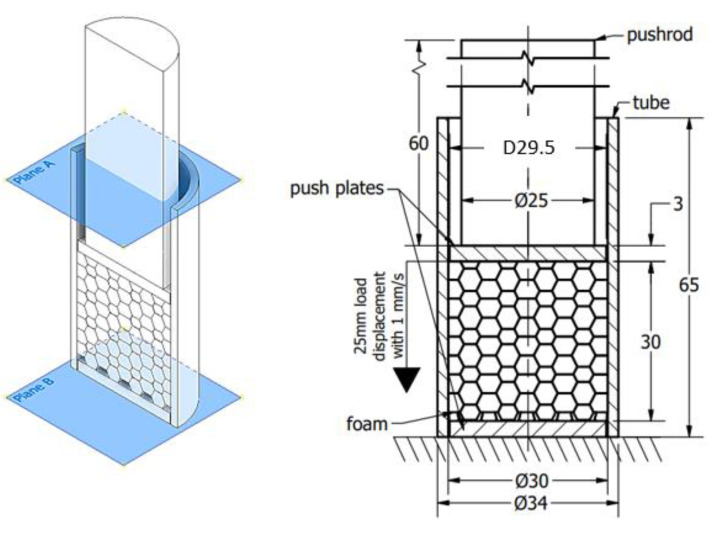
The scheme of radial-constrained test (Plane-A and B help to understand the orientions).

The main goal of the present investigation is to reveal the effect of friction concerning the radial constraint between the foam and tube compared with the uniaxial (without radial constraint) test. First, the foam was loaded using the crosshead without any radial inhibitor, resulting in a uniaxial compressive test. As a continuation of the test, the foams were inserted into the above-mentioned aluminium tube. The scheme of the radial constrained version can be seen in Figure 3.

The nominal diameter of foam specimens is equal to the inner nominal diameter of the tube and pushing plate, but to reveal the type of fit between them the real dimensions must be considered. Taking into account the real diameters, the transition fit occurred between the foam and the tube; so, to make this fit, a slight assembling force had to be applied on the foam. Concerning the fit of the pushing plate and tube clearance, the type of fit was applied to not influence the friction measurement between the foam and wall of the tube during the compression test. In the case of the pushrod, the type of fit does not make sense, since the nominal size of them is not the same. In order not to influence the measurement results, no lubricant was used for the assembly or during the compression test.

## 3. Radial-Constrained and Friction Test

### 3.1. Steps of Radial-Constrained Test

**Figure 4:**
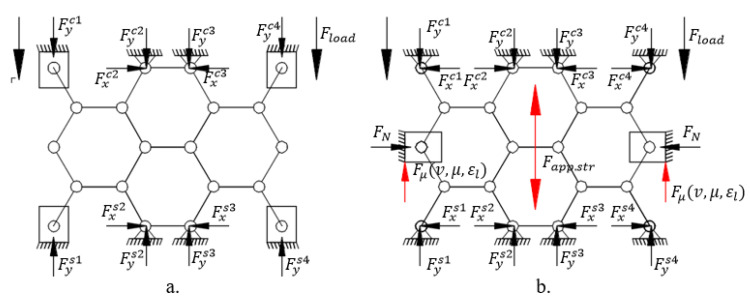
Representation of hinged structure. (**a**) free compressive, (**b**) radial-constrained.

During the test, the foam is loaded using axial compressive force inside the aluminium tube. Considering the Poisson effect of materials, the compression or tensile load is accompanied with radial strain [36]. In this case, the compression caused by radial strain is inhibited by the wall of the tube. Considering the closed foam structure as a 2D hinged structure, the DoF of its lateral node is blocked in the direction of the y-axis, increasing the stiffness and strength of construction. The simplified structures with nodes can be seen in Figure 4, where $F_{y}^{c}$. is the force of the compressive load by the node, $F_{y}^{s}$. is the supporting or reaction force, $F_{N}\;\mathrm{is}\;\mathrm{the}\;$ radial normal force, whilst $F_{\mu }$ represents the friction force.

However, it must be taken into account as an apparent stiffness enhancement caused by the presence of the wall of the tube and the inhibition of radial size increase. Owing to the inhibition of lateral displacement, the load results in cell wall deformation require more energy during the axial displacement. The cell wall can be matched with as a bar of the 2D hinged structure. Due to the presence of friction resistance between lateral nodes and the wall of constraint, the compression requires even more energy. Furthermore, considering the Poisson effect and the scale of the contact surface between the foam and tube, the conditions of friction resistance change continuously, affecting the required load. It was important to reveal the volume of the energy requirement concerning overcoming this friction resistance during the radial constrained test.

### 3.2. Method of Friction Test

The friction investigation could be divided into three main stages. In the case of the first stage, both density foams (0.4 g/cm^3^ and 0.7 g/cm^3^) were loaded individually via compression with 25 mm crosshead displacement, namely, a free compression test occurred. The test was applied three times. In the follow-up, the foam specimens were inserted into a tube, and the load was placed inside the tube using the pushing rod and plate. The applied displacement was the same, numerically 25 mm. Comparing the results of these two tests, the absorbed energy surplus of the radial-constrained version can be expressed easily.

To obtain the friction-caused energy proportion of this absorbed energy surplus, the third test stage is required. This process contains more sub-steps that continuously modify the constraints of the structure. Considering the radially constrained test seen in Figure 3, five different sections were made using different strains concerning the foam. In the case of the first specimen, the foam was inserted into the tube and non-loaded with any compression force. In the case of the second specimen, the foam is compressed with a 5 mm displacement, resulting in a 25 mm height. The third specimen is compressed with a 10 mm displacement to obtain a 20 mm height. The fourth and fifth ones are compressed with 15 and 20 mm, respectively. After the compression loads, the pre-compressed specimens are rotated and, using a push plate and pushrod, the foam is pushed away inside the tubes whilst the displacement is recorded to obtain information about the friction resistance.

**Figure 5:**
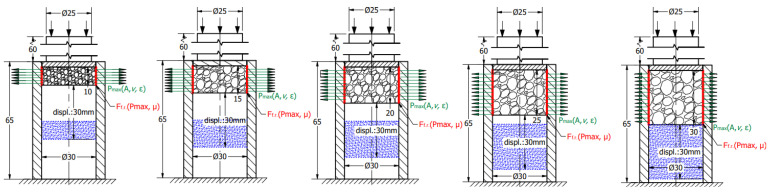
Scheme of friction measurement of pre-compressed foams.

Considering the five different compression states—owing to the Poisson effect—the radial/normal force ($P_{max}$) between the tube wall and the foam gets higher and higher whilst the area of the contact surface is modified, influencing the friction resistance during the foam displacement inside the tube. The concepts are introduced in Figure 5. The meaning of the colours is next. The green vectors describe the radial normal force ($P_{max}$) generated by the Poisson effect. The direction of vectors shows the direction of radial force loading the wall of the tube, whilst the length of them represents the magnitude of this normal force. The red lines indicate the friction force which is influenced by the $P_{max}$ and friction coefficient (μ). The blue hatched area symbolises the final position of the pre-compressed foam after they were pushed away inside the tube with a 30 mm displacement.

## 4. Friction and Contact Surface Issue

### 4.1. Friction Phenomena

**Figure 6:**
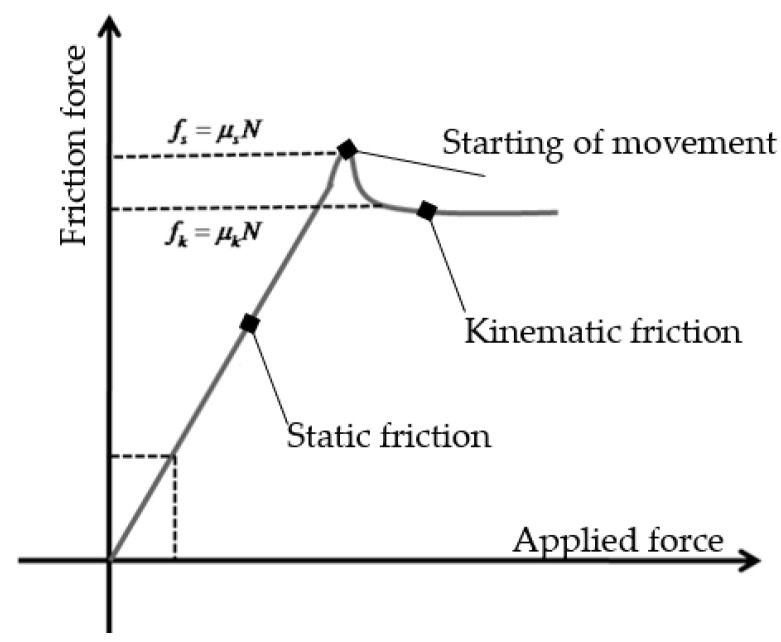
Graph of theoretical friction force in function of applied force.

Friction is one of the most interesting resistances in the field of engineering since sometimes it must be avoided, but sometimes, without its presence, the process cannot occur [37,38]. The friction is the resistance of the movement that occurs between the objects. The values strongly depend on more parameters like surface roughness, normal force, the speed difference between the contact surfaces, and the presence or lack of the lubricator. The friction force can be expressed using the well-known Coulomb Formula (1) [39,40].

(1) $$F_{fric.}=\mu \;\cdot \;F_{N}$$

where *μ* is the friction coefficient, whilst $F_{N}$ is the normal force between the connected objects. Physics fundamentals include the ideas of static and kinetic friction, and during the present test, both of them must be considered. Kinetic friction happens between surfaces that are moving, whereas static friction is the force that stops an item from moving when a force is applied [41,42,43]. While the coefficient of kinetic friction affects kinetic friction, the coefficient of static friction determines the amount of static friction concerning the normal force. Static friction often outweighs kinetic friction. The theoretical graph of the friction issue is introduced in Figure 6.

### 4.2. Contact Surface Calculation

**Figure 7:**
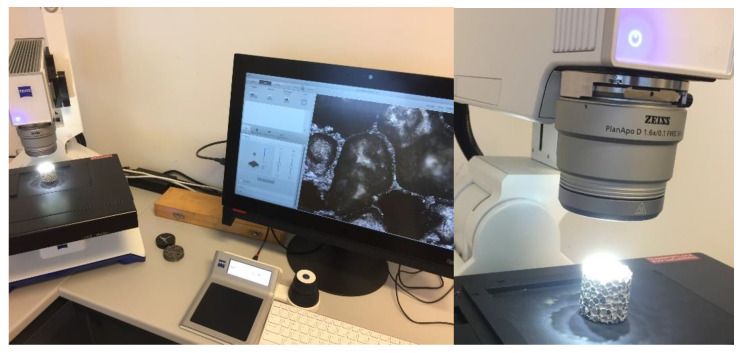
Microscope investigation to reveal the size of void inclusions and their deviations.

According to the science of friction, the contact surface size is not a crucial parameter during the investigation of friction issues. According to Figure 5, in this case, the normal force ($P_{max}$) and the size of the contact area are also changed continuously, affecting the friction resistance and influencing the energy requirement for the movement of pre-compressed foam. Due to the porous structure and surface of metal foams, it is not easy to define the exact area of contact surfaces of specimens inside the tube. To be informed about the scale of the contact area of cylindrical specimens, we performed microscopic analysis. For the image acquisition, the Zeiss Smartzoom 5 smart digital microscope was used with its own official Zeiss Zen v3.8 core software, presented in Figure 7.

**Figure 8:**
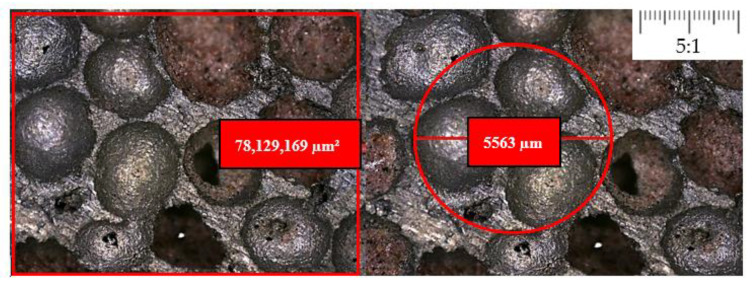
Microscopic view of the surface of foam specimen before the test.

**Figure 9:**
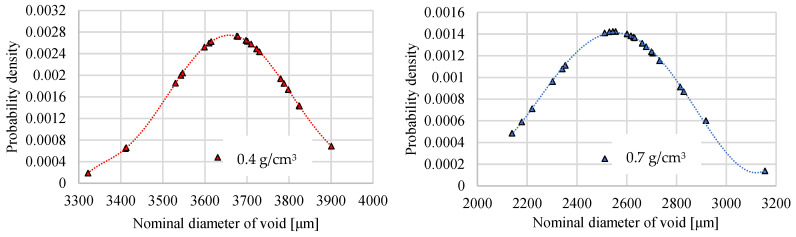
The Gauss curve around size of void inclusions of each foam.

Using the built-in digital measurement tools of the microscope, it is easy to measure the size of void inclusions and calculate the average distribution density. The print screen of the microscopic view with the dimension measuring tool is in Figure 8. Both density-type foams were subjects of the microscope measurements and presented the standard deviation in the Gauss diagram, whose results can be seen in Figure 9. The average void inclusions are 3680 µm and 2570 µm in the case of 0.4 g/cm^3^ and 0.7 g/cm^3^ density foam, respectively.

**Figure 10:**
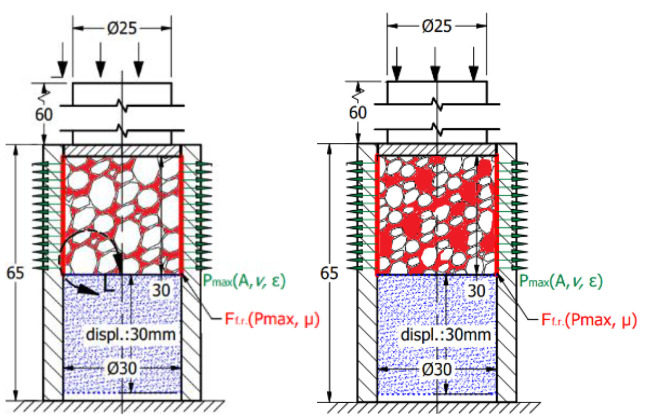
Representation of contact surface with different density foam.

To reveal the size of the contact surface between the foam and tube wall, a 100 mm^2^ unit area was first taken into account, where the number of cells can be easily to counted using a digital microscope and then extended for the whole surface of the cylindrical specimen, considering the aforementioned and detailed standard deviations. According to this method, 14.6 cells can be accounted for per unit of 100 mm^2^ area in the case of 0.7 g/cm^3^ type foam, and 8.3 cells in the case of 0.4 g/cm^3^ foam. Applying this calculation to the entire casing of the cylindrical test specimen, the contact area is the sum of the wall thicknesses between the inclusions on the surface. In the case of the 30 mm in diameter and height cylindrical solid specimens, they would have a 9.42 cm^2^ surface. Considering the number, size and deviation of void inclusions, the outer contact surface of foam is 2.42 cm^2^ in the case of denser foam, and 1.13 cm^2^ in the case of 0.4 g/cm^3^ type foam. These values are considered in the case of foams before the loading. Compressing the foam results in more and more cell deformation involving contact surface modification. The representation of this contact surface issue by the 0.4 and 0.7 g/cm^3^ foam is introduced in Figure 10, painted in red.

## 5. Evaluation Criteria

More evaluation criteria were used during the investigation to analyse the effect of the radial constraint and the friction between the foam and tube. The present study is a part of vehicle crashworthiness safety development; therefore, the absorbed energy by the foam is a focus area, as used for evaluation criteria. To obtain the absorbed energy by the specimens, the area under the force–displacement curve must be considered using graph integration (2), with Joules as the units of the values [44,45,46]. Concerning the closed-cell aluminium foam force–displacement test, the diagram can be divided into three main sections, namely the linear elastic section, plateau stage and densification zone [47,48,49,50]. Referring to this period, the absorbed energy is determined in the function of displacement to reveal the absorbing behaviour of each section as well.

(2) $$EA=\int F\left(x\right)dx\;\left[J\right]$$

From the point of view of vehicle crashworthiness safety development, weight optimisation is another crucial requirement; therefore, *SEA*, defined as Special Energy Absorbing, occurred, as one of the most frequently applied evaluation criteria in this research field (3) [51,52]. To determine this value, the ratio of absorbed energy and mass of specimens was calculated. However, according to another view, not only is the mass a crucial criterion, but the volume of energy absorber components of the crumple zone is also. Naturally, a larger crumple zone could involve higher absorbing capacity, but this scale is strongly limited by the required aesthetic design and shape. Unduly enlarged crumple elements take space away from other important components such as coolers, inverters, and control units, and can significantly reduce the car’s payload storage space [53]. Considering this issue, the definition of Volume Fraction (4) is used as a kind of useful complement or alternative to the frequently used *SAE*.

(3) $$SEA=\frac{\int F\left(x\right)dx}{m}\;\left[\frac{J}{g}\right]$$

(4) $$VF=\frac{\int F\left(x\right)dx}{V}\;\left[\frac{J}{cm^{3}}\right]$$

where $\int F\left(x\right)dx$ is the absorbed energy of the relevant zone, m is the mass, and V is the volume of the foam specimen. Using the Crashing Force Efficiency as an evaluation criterion, the ratio of the first maximum force and the mean value of the plateau zone can be expressed (4). Regarding the interpretation of CFE, the value is accounted in units of percent, and the main goal is to reach higher and higher efficiency [54,55].

(5) $$CFE=\frac{F_{avrg.}^{plateau}}{F_{max}}\;\left[\%\right]$$

where $F_{avrg}^{plateau}$ is the average resistance force of foam during the compression, whilst the F_max is the first maximum force of compression. The closed-cell aluminium foam could be a useful energy absorber in the crumple zones of vehicles, where the consistent absorbing behaviour is one of the most important requirements. Using the Crashing Force Fluctuation is suitable for analysing the assessment compliance with this criterion focusing on the section of plateau. The CFF  is accounted as the ratio between the real absorbed energy of the plateau zone and the mean force-based absorbed energy of the plateau zone (6). This evaluation consideration is frequently named ULC—Undulation of Load Carrying Capacity [56,57,58].

(6) $$CFF=\frac{\int \left|F\left(x\right)-F_{m}\right|dx}{\int F\left(x\right)dx}$$

where $\int \left|F\left(x\right)-F_{m}\right|dx$ is the energy different considering the real and mean of crushing force, while $\int F\left(x\right)dx$ is the real absorbed energy.

## 6. Results of Uniaxial Load

**Figure 11:**
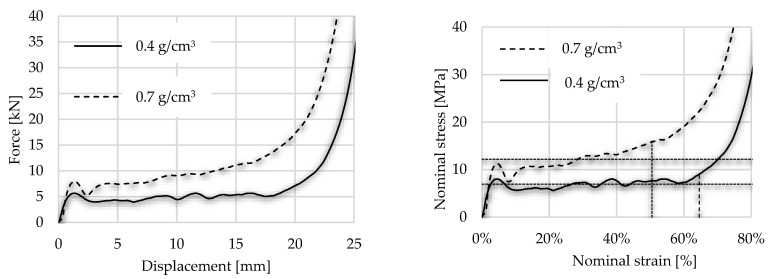
Displacement force and stress–strain curves of free compression test.

As the first step, the foams were loaded by 1 mm/s without any type of radial inhibitor accessories. The foam graph followed the typical force–displacement diagram with linear elasticity, plateau and densification. To obtain a stress–strain curve, compressive force must be divided by the initial cross-sectional area perpendicular to the loading direction. The effect of foam density can be considered; concerning the first maximum force, lighter (0.4) and denser (0.7) foam have 5.66 kN and 7.94 kN, respectively, as the first local maximum in the force–displacement curve. During the test, the ISO 13314 (2011) standard is taken into consideration, according to which the plateau stress is the arithmetical mean of the stresses at 0.1% or smaller strain intervals between 20% and 30% or 20% and 40% compressive strain [59]. The mean value of plateau stress was less with 15% in the case of lighter foam than the denser one. Numerically, the average stress in the plateau zone was 6.92 MPa concerning lighter ones and 12.26 MPa in the case of denser. The end of the plateau zone is defined by the above-mentioned ISO standard as the point in the stress–strain curve at which the stress is 1.3 times the plateau stress. Considering this rule, it is easy to define the densification point of the specimen, numerically 64.28% and 50.53% regarding the lighter and denser foam, respectively. The graphs of the free compression tests are presented in Figure 11, where the vertical dotted lines indicate the end of plateau.

## 7. Results of Radial-Constrained Test

**Figure 12:**
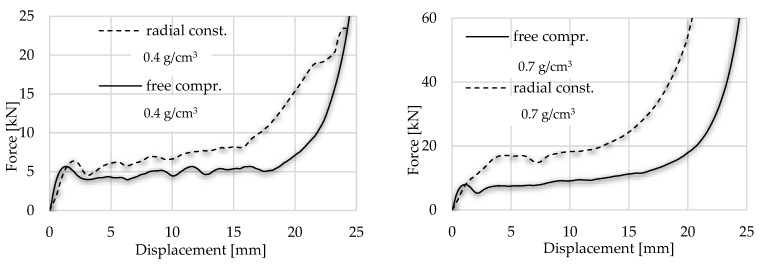
Displacement force diagram to compare the free and radial-constrained compressive tests.

The hypothesis according to which the radially constrained foam results in a higher energy level during its compressive load is confirmed by the present investigation as well. The typical sections such as linear elastic zona, plateau and densification can be observed in this test situation as well. However, the plateau zone has a shorter length and a slight steepness compared with the uniaxial load version. During this compressive load, the force displacement shows higher characteristics, explained by the above-mentioned apparent strength increase and friction. Numerically, the plateau zone in this case can be characterised with 24.48 MPa in the case of denser foam, while with the lighter foam, this value was 9.51 MPa. Another significant deviation between the two concepts was the densification strain. The results of the radially constrained test are in Figure 12 and are compared with the uniaxial load.

## 8. Absorbed Energies and Efficiencies

Considering the energy levels, the 0.4 g/cm^3^-type foam in the radial-constrained version was able to absorb 32.5% more energy during the compression compared with the uniaxial load concept. The 0.7 g/cm^3^ foam called for 90.5% more compression energy during the radial-constrained test. Since the tube wall inhibited the radial deformation of cells of foam specimens, the end of the plateau zone in these cases occurred earlier. Numerically, the 0.4 gm/cm^3^ and 0.7 g/cm^3^ foam have plateau stress with 7.8 MPa and 14.5 MPa, respectively. Considering the above-mentioned ISO standard, it is easy to define the end of the plateau since it is 1.3 times of plateau value. The end of the plateau is the starting moment of the densification zone, which occurred at a strain of 64.28% and 53.86% in the case of 0.4 g/cm^3^ foam during the free and radial-constrained test, respectively. With the same conditions, the appearance of densification of the denser foam was 50.53% and 47.19%. Analysing the crashworthiness behaviour of the absorber, the plateau zone has a dedicated role; therefore, the absorbed energies and efficiencies are calculated concerning the plateau zone as well. The plateau zone and densification issues are collected in Table 2. The SAE and VF values are not relevant for the foam in the case of the radially constrained test, since in that case the weight and volume of the aluminium tube and the accessories ought to be taken into account as well. The results of the present investigation could be also validated by the study of Yang et al. where different density types of closed-cell aluminium foams were tested and confirmed the values of 0.43 and 0.7 foam as well [60].

**Figure 13:**
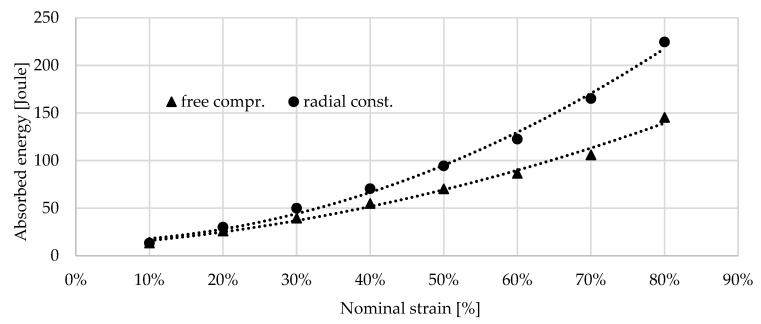
Absorbed energy by 0.4 g/cm^3^ foam between 10 and 80% nominal strain.

**Figure 14:**
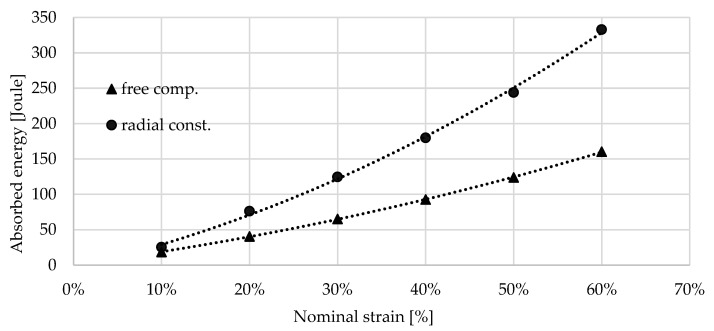
Absorbed energy by 0.7 g/cm^3^ foam between 10 and 60% nominal strain.

As it was mentioned above, the loading was limited at 80 kN due to the prevention of the overloading of the load cell; therefore, the radially constrained compression test of the 0.7 g/cm^3^ foam was able to run up to a strain of 66.29% which only corresponds to the displacement of 19.88 mm. The energies are collected in Table 3 and Table 4; these are presented in Figure 13 and Figure 14.

## 9. Results of Friction Measurement

As the description above confirmed, the radially constrained condition for foam involves a higher compressive energy requirement due to the inhibited cell deformation and the overcoming of friction between the foam and the wall of the tube. The main goal of this study is to reveal the friction proportion during the radial-constrained load. The steps of the friction measurement are introduced in Section 3.2.

Firstly, the 0.4 g/cm^3^ foam was partially loaded by 5 mm compression displacement. Then, the pre-compressed foam was pushed via the tube from the other direction via the tube with 30 mm of displacement whilst the force–displacement curve was recorded to obtain a friction chart about the movement. The measurement was continued by pre-loading another foam specimen with a displacement of 10 mm and then pushing it through the tube from the other direction with a displacement of 30 mm. This step was followed with 15 and 20 mm pre-loading and friction measurements. Naturally, the process was repeated, involving 0.7 g/cm^3^ foam as well.

**Figure 15:**
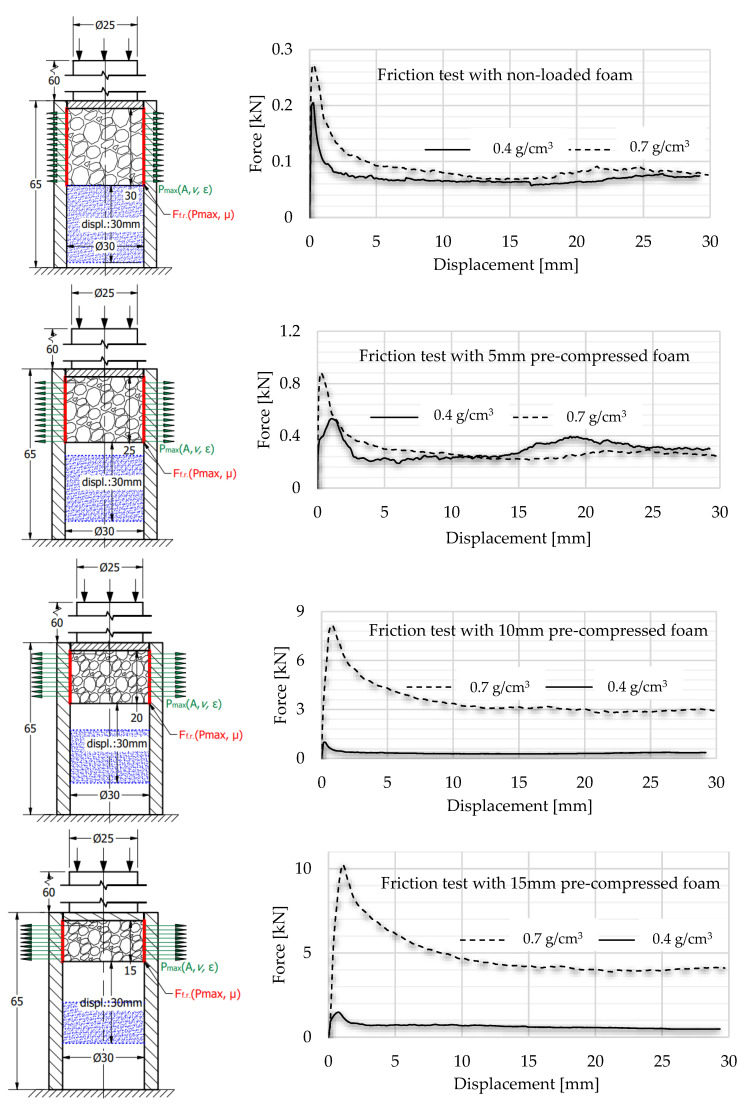

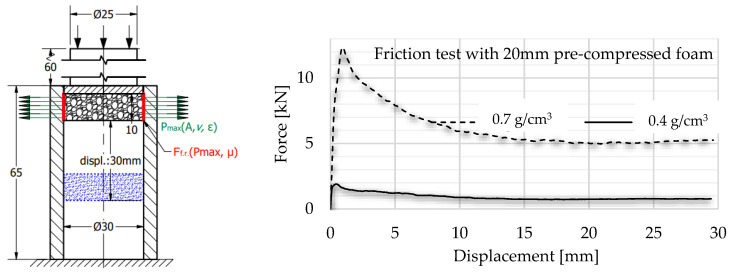
Friction graphs of each pre-compressed specimen pushed away inside the tube.

**Figure 16:**
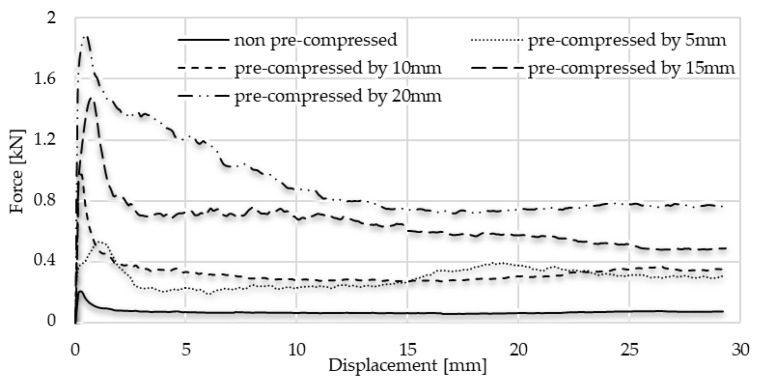
Comparison of friction graphs of 0.4 g/cm^3^ foam.

**Figure 17:**
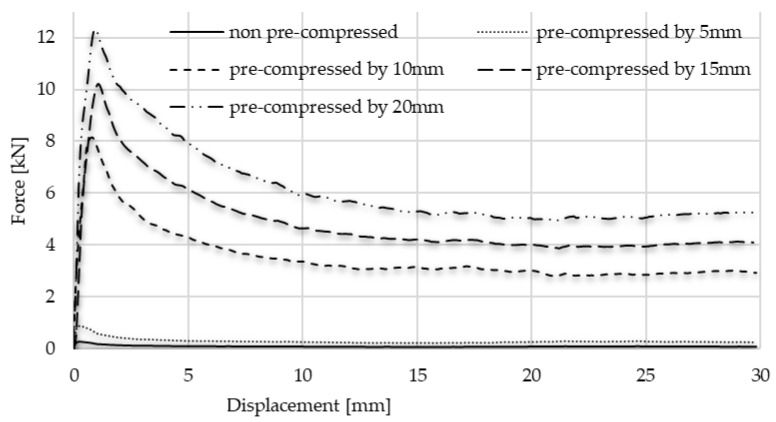
Comparison of friction graphs of 0.7 g/cm^3^ foam.

**Figure 18:**
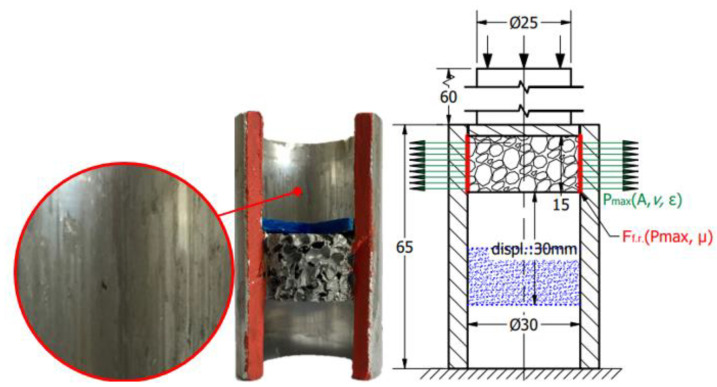
Section view after 15 mm pre-compressed foam friction measuring.

According to the measurement, the graph follows the typical static–kinematic form of a diagram. The results of the test confirmed the initial hypothesis, according to which the notable part of the energy surplus occurred in the radially constrained test caused by friction. Through analysing the graphs, the typical kinematic and static resistance can be obtained. When the 0.4 g/cm^3^ foam was pre-compressed with 5 mm displacement, 0.51 kN force was applied to move it in the tube, and then the kinematic friction prevailed and called for a 0.29 kN moving force. Observing these values regarding pre-compressed foam with 10 mm, the kinematic resistance was 0.32 kN, whilst the static value of it was 0.97 kN. In the pre-loaded foam using 15 mm compression, the static resistance required 1.47 kN force, whilst the kinematic result showed 0.69 kN. In the case of the 20 mm-pre-loaded foam, the static resistance was 1.85 kN, and the kinematic resistance was 0.91 kN. And naturally, it is important to know the friction of foam without pre-load. In this condition, the movement required 0.16 kN force to overcome the static friction and 0.06 kN force to keep it moving. Then, the friction investigation steps were repeated, related to the 0.7 g/cm^3^ foam as well. As was expected, the friction resistances were significantly higher, since the connection area of specimens is larger. This surface phenomenon is confirmed using the void-area calculation via the smart microscope view detailed in Section 4. The test includes 5–10–15–20 mm pre-compression where the static frictions were 0.87 kN; 7.89 kN; 10.05 kN; and 12.21 kN, respectively. The movement of the non-pre-loaded specimen called for 0.53 kN force to overcome the static friction. The forces required to keep the test specimens in motion to exceed the kinematic friction are, respectively: 0.26 kN; 3.25 kN; 4.53 kN; and 5.77 kN. In the case of the non-pre-loaded version, the foam movement required 0.08 kN. The results of the friction measurement step-by-step are presented in Figure 15, whilst Figure 16 and Figure 17 summarise the graphs of each foam. Table 5 is about the collection of static and kinematic friction resistance in the function of pre-loading specimens. After the pre-compressed foam is pushed away inside the tube, the worn, scratched surface is well-observed. A section view was made about the 15 mm pre-compressed foam after the friction, measuring to show the surface roughness and the final position of the foam. The section view is presented in Figure 18 and is compared with the initial sketch drawing.

**Figure 19:**
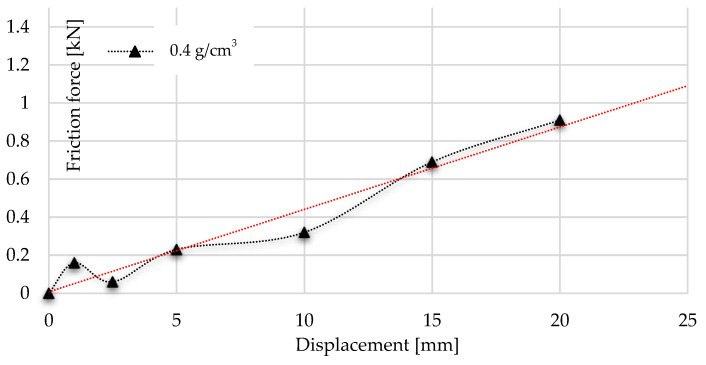
Trend of frictional resistance during multiaxial test using foam with a density of 0.4 g/cm^3^ with trendline.

**Figure 20:**
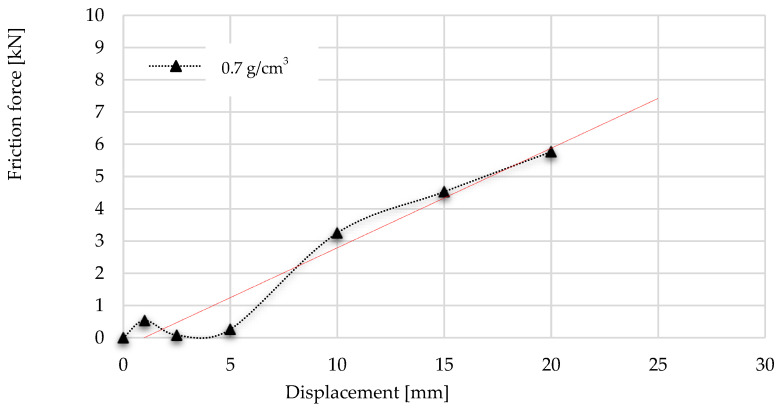
Trend of frictional resistance during multiaxial test using foam with a density of 0.7 g/cm^3^ with trendline.

Considering the results of the above test, the friction graph of the radial-constrained tests can be constructed using the values of kinematic friction forces and the initial static friction force. These graphs are introduced in Figure 19 and Figure 20. The diagrams start with the first local maximum presenting the overcoming of static friction, then are followed by the kinematic friction resistance, which can be observed. However, due to the inner compression of the foam, the radial force ($P_{max}$) between the foam and the tube wall increases continuously, which is manifested in an increasing kinematic friction characteristic. The radial compression ($P_{max}$) increase issue is presented in Figure 5, indicated by green arrows. Using the linear trend line, the friction resistance can be predictable as a function of further displacement. The usefulness of the friction functions is that they can be used to determine how much energy is required to overcome the frictional resistance arising during the radially inhibited compaction. To define this energy, the integration of friction graphs is required, with up to 25 mm displacement; these are then divided into different ranges.

According to the integration of friction graphs, the radial-constrained compression of 0.4 g/cm^3^ foam required 0.6 J of energy to overcome the friction up to the displacement of 5 mm. Above 5 mm, due to the increase in radial force ($P_{max}$) of foam, 1.98 J was needed to overcome the friction resistance. Going on with the displacement, 4.51 J of energy was required to keep moving the object and overcome the kinematic friction between the foam and tube wall. Summarising the energy requirement up to 20 mm, 8.52 J was needed to overcome the friction. In the case of 0.7 g/cm^3^ foam, the radially constrained concept required 28.31 J more energy up to the 5 mm displacement. Of this energy surplus, 1.08 J was used to overcome friction, whilst 9.86 J of energy surplus was needed up to 10 mm displacement owing to the presence of resistance. A total of 28.82 J of energy was required in the case of a radial-constrained version of 0.7 g/cm^3^ foam to exceed the friction resistance during the 0–15 mm displacement. Considering the 0–20 mm displacement period, the sum of the friction energy demand was 53.98 J. Table 6 and Table 7 summarise the results of energy requirements.

## 10. Discussion

The compression test is a useful investigation form to reveal the mechanical behaviour of aluminium foam under loading, such as plateau stress, energy absorbing capacity and energy absorbing efficiencies, which are crucial evaluation considerations during crashworthiness development. Many studies are about free compression tests of metal foams, but the number of investigations about radial-constrained loads is moderate. The foam compression under radial constrain demands significantly more energy. This energy surplus is caused by the friction between the foam and the wall of constraint and the inhibition of radial deformation of the specimen. However, in the literature, there is no information about this friction resistance during the radially constrained test; therefore, the main goal of the present study was to reveal this friction resistance. Considering the results of the present investigations, the next conclusion can be stated.

Using the CFE (Crushing Force Efficiency) and CFF (Crushing Force Fluctuation) as the evaluation criteria to analyse the mechanical and folding behaviour of foam during radial-constrained loading is not relevant, since they show more than 100% due to the steepness of the end of the plateau zone; therefore, they can result in misunderstanding and deceptive results during the analysis. Instead of these conditions, the analysis must focus on the energy levels and strain of densifications. The CFE  and CFF  relevant values are collected in Table 2.

In this study, to obtain a relevant comparison between free and radial-constrained tests, the ISO13314:2011 standard was applied and taken into consideration. The standard characterises the method of calculation of plateau stress, plateau zone and the starting strain of densification concerning the free compression test, but there is no determination of conditions of the radial-constrained load, materials and friction parameters. Therefore, further friction tests were necessary to obtain a meaningful answer on the effect of radial forcing.

The foam with a density of 0.4 g/cm^3^ absorbed an average of 31.2% more energy during the radially constrained compressive test compared to the free compressive one. The plateau range is a particularly important period of foam compression investigation; therefore, the difference in absorbed energy was also determined until the end of the plateau zone. In this case, the radially blocked foam absorbed 93.96 J, while the radially inhibited case absorbed 104.33 J, which means an 11.1% increase. Based on these, it can be stated that the significant energy surplus occurs in the densification zone and not in the plateau.

The test ranges were divided into different ranges by 5 mm displacements, such as 0–5 mm; 0–10 mm; 0–15 mm; and 0–20 mm. The total absorbed energy and the energy due to friction resistance are accounted for in all ranges. Based on the values via friction tests, the initial hypothesis has been confirmed, according to which the Coulomb friction during the compression inside a tube is influenced by the strain of the foam, the size of the contact surface between foam and constrain, and the Poisson ratio of the foam. Considering the values of Table 6, it can be stated that 18% of the invested energy was used to overcome friction in the case of the radial-constrained foam compression test.

The free compression test of 0.7 g/cm^3^ foam required 126.53 J up to the end of the plateau zone, which is less by 44% compared to the radial-constrained version. Numerically, the constrained version demanded 225.31 J of energy before the beginning of the densification zone. The energy difference is 98.78 J, 39.64% of which was used exclusively to overcome the friction between the tube wall and the foam.

The measurement proved that the foam with a higher density can absorb more energy per unit displacement, but taking into account the SEA value introduced due to mass optimisation, it can be seen that the foam with a density of 0.4 g/cm^3^ proves to be more efficient. Therefore, it is important to express how much energy a unit mass of foam can absorb per unit displacement. Based on these, it can be stated that if the goal in the design of the folding structure is to achieve a higher absorption capacity, then it is not the goal to use metal foam with a higher density in the structures. To reveal more information about the friction between the foam and tube, a surface roughness investigation is suggested to consider the wall of the tube before and after the test. Furthermore, an inner-lubricated version could provide useful information about the friction resistance proportion during the radial-constrained foam test.

## 11. Conclusions

The present work investigates the friction phenomena of a closed-cell aluminium foam-filled Al tube through compression testing. Between 0.43 and 0.7 g/cm^3^ density foam was used in the same test conditions. This research could be filling in a gap in the field of foam-filled crash box structures science, since it reveals the magnitude of friction during a radial-constrained metal foam compression test. The novelty of the present research is not only in the values, but also in the applied method used during the investigation.

The radial-constrained compression test of 0.43 g/cm^3^ foam calls for more energy with, 53.6% more than the free compression type. An average of 16% of this extra energy is due to the friction between the pipe wall and the foam. Considering the results of the 0.7 g/cm^3^ foam, when the foam was compressed inside the aluminium tube, the process required about two times more compression energy than the free compression version. However, 23% of this energy surplus occurred owing to the friction phenomena.

Under compression, increasing the density of aluminium foam leads to a shorter plateau zone. The mechanical characteristics of aluminium foam can be greatly enhanced by adding a desirable gradation in pore frequency and altering the pore distribution. For foams with a relative density of 0.7, this can increase the plateau stress by nearly 100% and improve energy absorption as well. Since aluminium foam’s relative density and compressive strength and Young’s modulus are related, aluminium foam’s density plays a critical role in determining how it behaves mechanically. A higher density of aluminium foam typically translates into increased stiffness, strength, and capacity to absorb energy. However, according to the results of the present study, the authors state that it is more appropriate to choose foam with a lower density than 0.7 g/cm^3^ for radially inhibited compaction, as the foam quickly reaches the densification zone during compaction, especially in the case of the radially inhibited version, and thus its distorting and misleading results have an effect on energy and friction tests. However, it was a useful comparison, since it was observed that in a case of higher relative density, the presence of friction phenomena during the radial compression test is more intensive.

## Figures and Tables

**Table 1:** Dimensions of specimens.

Designation of Specimens	Lighter Foam			Denser Foam		
	Mass [g]	Volume [cm^3^]	Density [g/cm^3^]	Mass [g]	Volume [cm^3^]	Density [g/cm^3^]
SP1	9.24	21.21	0.43	14.25	21.19	0.67
SP2	9.20	21.17	0.43	14.05	21.19	0.66
SP3	9.31	21.09	0.44	14.31	21.32	0.67
Average	9.25	21.15	0.43	14.20	21.26	0.67

**Table 2:** Plateau range relevant results.

Results	Free Compr. Test of 0.4 g/cm^3^	Radial Const. Test of 0.4 g/cm^3^	Free Compr. Test of 0.7 g/cm^3^	Radial Const. Test of 0.7 g/cm^3^
Plateau stress (20–40% strain) [MPa]	6.92	9.51	12.26	24.48
Nominal strain of end of plateau [%]	64.28	53.86	50.53	47.19
Stroke of end of plateau [mm]	19.28	16.15	15.16	14.16
EA up to end of plateau zone [J]	93.96	104.33	126.13	225.31
SEA up to end of plateau zone [J/g]	10.15	not relevant	8.88	not relevant
VF up to end of plateau zone [J/cm^3^]	13.31	not relevant	17.86	not relevant
CFE up to end of plateau zone [%]	77.91	106.13	108.43	112.54
CFF of plateau zone [%]	0.15	3.8	1.11	29.61

**Table 3:** Absorbed energy by 0.4 g/cm^3^ foam in each period given in Joules.

Stroke [mm]	Free Compression Test	Radial-Constrained Test	Differential	
0–5	20.87 J	23.86 J	2.99 J	+11.4%
0–10	43.48 J	53.37 J	9.89 J	+22.7%
0–15	68.88 J	94.42 J	25.54 J	+37.1%
0–20	97.51 J	149.79 J	52.28 J	+53.6%

**Table 4:** Absorbed energy by 0.7 g/cm^3^ foam in each period given in Joules.

Stroke [mm]	Free Compression Test	Radial-Constrained Test	Differential	
0–5	31.53 J	59.84 J	28.31 J	+89.7%
0–10	72.21 J	142.93 J	70.72 J	+97.93%
0–15	121.54 J	244.03 J	122.49 J	+100.1%
0–20	190.03 J	415.18 J	225.03 J	+118.48%

**Table 5:** Friction resistances of different pre-compressed foam.

Foam Pre-Loading	Mean of Static Friction Force [kN]		Mean of Kinematic Friction Force [kN]	
	0.4 g/cm^3^	0.7 g/cm^3^	0.4 g/cm^3^	0.7 g/cm^3^
Non-pre-compressed	0.16	0.53	0.06	0.08
Pre-compressed by 5 mm	0.51	0.87	0.29	0.26
Pre-compressed by 10 mm	0.97	7.89	0.32	3.25
Pre-compressed by 15 mm	1.47	10.05	0.69	4.53
Pre-compressed by 20 mm	1.85	12.21	0.91	5.77

**Table 6:** Energy requirements by 0.4 g/cm^3^ foam given in Joules.

Stroke [mm]	Free Compr. Test	Radial Const. Test	Differential	Energy Requirement to Overcome the Friction
0–5	20.87 J	23.86 J	2.99 J	0.6 J
0–10	43.48 J	53.37 J	9.89 J	1.975 J
0–15	68.88 J	94.42 J	25.54 J	4.51 J
0–20	97.51 J	149.79 J	52.28 J	8.52 J

**Table 7:** Energy requirements by 0.7 g/cm^3^ foam given in Joules.

Stroke [mm]	Free Compr. Test	Radial Const. Test	Differential	Energy Requirement to Overcome the Friction
0–5	31.53 J	59.84 J	28.31 J	1.08 J
0–10	72.21 J	142.93 J	70.72 J	9.86 J
0–15	121.54 J	244.03 J	122.49 J	28.22 J
0–20	190.03 J	415.18 J	225.03 J	53.98 J

## Data Availability

The original contributions presented in the study are included in the article, further inquiries can be directed to the corresponding author.
